# Human epidermal growth factor receptor-2 expression and subsequent dynamic changes in patients with ovarian cancer

**DOI:** 10.1038/s41598-024-57515-y

**Published:** 2024-04-05

**Authors:** Yoo-Na Kim, Yun Soo Chung, Eunhyang Park, Seung Tae Lee, Jung-Yun Lee

**Affiliations:** 1 Department of Obstetrics and Gynecology, Institute of Women’s Life Medical Science, Yonsei University College of Medicine, 50-1 Yonsei-ro, Seodaemun-gu, Seoul, 03722 Korea; 2 Department of Pathology, Yonsei University College of Medicine, Seoul, Korea; 3 Department of Laboratory Medicine, Yonsei University College of Medicine, Seoul, Korea

**Keywords:** Targeted therapies, Tumour biomarkers, Ovarian cancer

## Abstract

Human epidermal growth factor receptor-2 (HER2)-targeting drugs are increasingly being incorporated into therapeutic paradigms for non-breast cancers, yet studies on HER2 expression in ovarian cancer (OC) are inadequate. Here, we studied the HER2 status and dynamic changes in OC by reviewing the records of patients who underwent HER2 testing at a single institution. Clinical parameters, including histology, *BRCA* status, and immunohistochemistry (IHC), were evaluated alongside HER2 expression, timing, and anatomical location. Among 200 patients, 28% and 6% exhibited expression scores of 2+ and 3+, respectively. HER2 3+ scores were observed in 23%, 11%, 9%, and 5% of mucinous, endometrioid, clear cell, and high-grade serous tumors, respectively, and were exclusively identified in *BRCA*-wildtype, mismatch repair-proficient, or PD-L1-low-expressing tumors. The *TP53* mutation rate was low, whereas *ARID1A, KRAS,* and *PIK3CA* mutations were relatively more prevalent with HER2 scores of 2+ or 3+ than with 0 or 1+. Four of the five tumors with an HER2 3+ score exhibited *ERBB2* amplification. Among 19 patients who underwent multiple time-lagged biopsies, 11 showed increased HER2 expression in subsequent biopsies. Patients with HER2-overexpressing OC exhibited distinct histological, IHC, and genomic profiles. HER2-targeting agents are potential options for *BRCA*-wildtype patients, particularly as later lines of treatment.

## Introduction

Ovarian cancer (OC) stands as one of the most fatal gynecological malignancies, posing challenges regarding early diagnosis and frequent relapses in response to first-line chemotherapy^1,2^. Consequently, the use of targeted therapy based on immunohistochemistry (IHC) or next-generation sequencing (NGS)-based profiling has been suggested^3–5^. Human epidermal growth factor receptor-2 (HER2), a proto-oncogene encoding a tyrosine kinase receptor, is one of the most extensively studied targets in breast cancer^6–8^. The exploration of HER2-targeted therapies beyond breast cancer has gained traction subsequent to their establishment as the standard of care for breast cancer^9^. Phase II trials involving early generation anti-HER2 molecules, such as trastuzumab and lapatinib, have demonstrated minimal response rates in heavily treated patients with OC^10,11^. In the recent National Cancer Institute Molecular Analysis for Therapy Choice trial involving trastuzumab emtansine treatment, three patients with OC exhibited stable disease for > 6 months^12^. Moreover, a phase II basket trial involving trastuzumab deruxtecan (T-Dxd) treatment in locally advanced or metastatic setting (DESTINY-PanTumor02) revealed an outstanding objective response rate (ORR) across multiple cancers. The response was particularly poignant among tumors with HER2 3+ scores, suggesting a potential for T-Dxd to serve as a tumor-agnostic biomarker-driven therapeutic option^13^.

Despite these advancements in HER2-targeted therapy, previous research on HER2 in the context of OC has primarily focused on their positivity rates and association with prognostic factors. Moreover, differences in specimen type^14^ and sampling timing, before and after exposure to neoadjuvant chemotherapy or targeted therapy^15^, have been reported. Regarding the correlation between HER2 expression and prognostic factors in OC, existing evidence suggests an association with poor histological grade, advanced stage, and shorter survival time^16^. However, the comparability of these trials across studies has been challenging owing to the limited number of patients and the aforementioned technical variations.

Given these constraints, this study was undertaken to investigate HER2 expression, incorporating various IHC and genomic biomarkers. Our emphasis centered on elucidating the clinical applicability of HER2-targeted therapy for OC in real-world settings. In this retrospective study, we identified all patients who underwent IHC testing for HER2 status and comprehensively assessed their clinical parameters, *BRCA* status, IHC biomarker status (mismatch repair [MMR] protein levels and programmed cell death ligand 1 [PD-L1] expression), and tumor NGS profiles. Moreover, in a subset of patients who underwent multiple testing, we investigated HER2 expression with respect to the anatomic localization of the tumor and delineated associated changes in the context of time-lagged biopsies.

## Methods

### Patient recruitment and sample acquisition

Patients with OC who underwent HER2 status testing between January 2015 and May 2021 were identified. Most of these patients were subjected to *BRCA* status assessments, IHC for biomarkers, such as MMR protein levels and PD-L1 expression, and tumor NGS, as part of OC management. This study was approved by the institutional review board of Yonsei University Severance Hospital (#4-2022-0247) and was performed in accordance with the principles in the Declaration of Helsinki. The requirement for informed consent was waived by the ethical committee of Severance Hospital owing to the retrospective nature of the study.

### IHC

Formalin-fixed paraffin-embedded (FFPE) tissue specimens were used for IHC. After deparaffinization with xylene and rehydration with graded alcohol, IHC was performed using Ventana Discovery XT Automated Slide Stainer (Ventana Medical System, Tucson, AZ, USA). The cell conditioning 1 buffer (citrate buffer, pH 6.0; Ventana Medical System) was used for antigen retrieval. The sections were incubated with the following primary antibodies for each IHC-based biomarker: anti-HER2 (1:1500; polyclonal; DAKO, Glostrup, Denmark), MLH1 (1:50; BD Biosciences, San Jose, CA, USA), MSH2 (1:200; BD Biosciences), MSH6 (1:100; Cell Marque Corporation, Rocklin, CA, USA), PMS2 (1:40; Cell Marque), PD-L1 (prediluted; clone SP263; Ventana Medical System), and anti-PD-L1 (1:50; clone 22C3; DAKO). HER2 expression was determined based on the HER2 scoring criteria for gastric cancers reported by Hofmann et al.^17^, and the interpretation criteria are shown in Table S1^15^. MMR protein status was considered aberrant if the tumor cells showed the complete absence of nuclear staining compared to a positive nonneoplastic internal control; an intact status was assigned if tumor cells displayed nuclear positivity^18^. Combined positive score (1:50, clone 22C3; DAKO) and tumor proportion score (prediluted clone SP263; Ventana Medical System) were calculated as previously described previously^19^ to determine PD-L1 expression. Positive PD-L1 expression was determined based on a combined positive score of ≥ 10. All IHC results were scored and interpreted by an expert pathologist (E Park).

### NGS analysis of tumor samples

Tumor samples were prepared from FFPE tissues. An expert pathologist reviewed the hematoxylin–eosin-stained slides to ensure adequate tumor content. For DNA extraction, two to five slides of resected specimens with a thickness of 5 μm each were used. FFPE samples with high tumor cellularity (> 10%) were subjected to NGS analysis.

Genomic DNA was extracted using a Maxwell CSC DNA FFPE Kit (Promega, Madison, WI, USA) according to the manufacturer’s instructions. The products were sequenced using the NextSeq 550 platform (Illumina, San Diego, CA, USA). Mutational and copy number analyses were performed using the TruSight Tumor 170 or TruSight Oncology 500 panels (Illumina). For mutational analysis, FASTQ files were uploaded to the Illumina BaseSpace software (Illumina) for variant interpretation. Only variants in the coding or promoter regions or splice variants were retained. In addition, variants present in 3% of the reads with a minimum read depth of 250 were retained. All retained variants were reviewed against reference websites [Catalogue of Somatic Mutations in Cancer (http://evs.gs.washington.edu/EVS/), Precision Oncology Knowledge Base (http://oncokb.org), and Database For Single Nucleotide Polymorphisms (https://www.ncbi.nlm.nih.gov/snp)]. Only the pathogenic variants were selected for further analyses. In the copy number analysis, only genes with more than a two-fold change in expression relative to the average level were considered for amplification. The NGS results, inclusive of clinical reports and filtered, annotated variant calling files delineating pathogenic somatic mutations, were used to determine somatic *BRCA* status and compile a list of somatic alterations.

### Acquisition of relevant gene lists

Actionable somatic alterations are defined as alterations that can be targeted by a drug available for on-label, off-label, or clinical trials. These alterations were selected based on a literature search of the MD Anderson Knowledge Base for Precision Medicine (http://PCT.MDAnderson.org) and TCGA (http://cancergenome.nih.gov/).

### Panel-based germline testing

Germline DNA was evaluated using a customized targeted capture sequencing panel (OncoRisk, Celemics, Seoul, Korea), covering all the coding sequences and intron–exon boundaries of the coding exons of 65 genes known to be associated with cancer predisposition, as previously described^20^. Structural and nucleotide variants were also evaluated. Germline variants were classified as pathogenic, likely pathogenic, and of uncertain importance and were reported in accordance with the guidelines of the American College of Medical Genetics and Genomics^21^. Only pathogenic or likely pathogenic germline variants of genes associated with cancer predisposition were considered in the analysis. The read-depth-based detection of structural variants was performed using ExomeDepth software^22^.

### Collection of clinical, IHC, and genomic variables

Data on clinical variables, such as histology, stage, platinum-free interval (PFI), and types of therapy received, were collected. The status of IHC-based biomarkers, such as HER2 and MMR protein levels and PD-L1 expression, was compiled. Germline and tumor NGS data, including the *BRCA* status, were reviewed for genomic profiles. For patients who underwent multiple HER2 testing, we investigated HER2 expression with respect to anatomic location and changes in cases of time-lagged biopsies. In instances of multiple HER2 tests on a single patient, the highest value was used, unless otherwise specified.

### Statistical analysis

Statistical analysis was performed using R version 4.0.3 (R Foundation for Statistical Computing, Vienna, Austria). The variant calling file from the aforementioned NGS pipeline was used for analysis and visualization with the “maftools” package in R. Significance was evaluated using Fisher’s exact test or chi-square test for categorical variables and the student’s *t*-test for continuous variables, where applicable. For all analyses, significance was set at P < 0.05 based on two-tailed test.

### Ethics approval and consent to participate

This study was approved by Yonsei University Severance Hospital’s institutional review board (#4-2022-0247) and was performed in accordance with the principles of the Declaration of Helsinki. The need for informed consent was waived by the ethical committee at Severance Hospital because of the retrospective nature of the study.

## Results

### Patient recruitment and HER2 expression with respect to clinical variables

**Figure 1:**
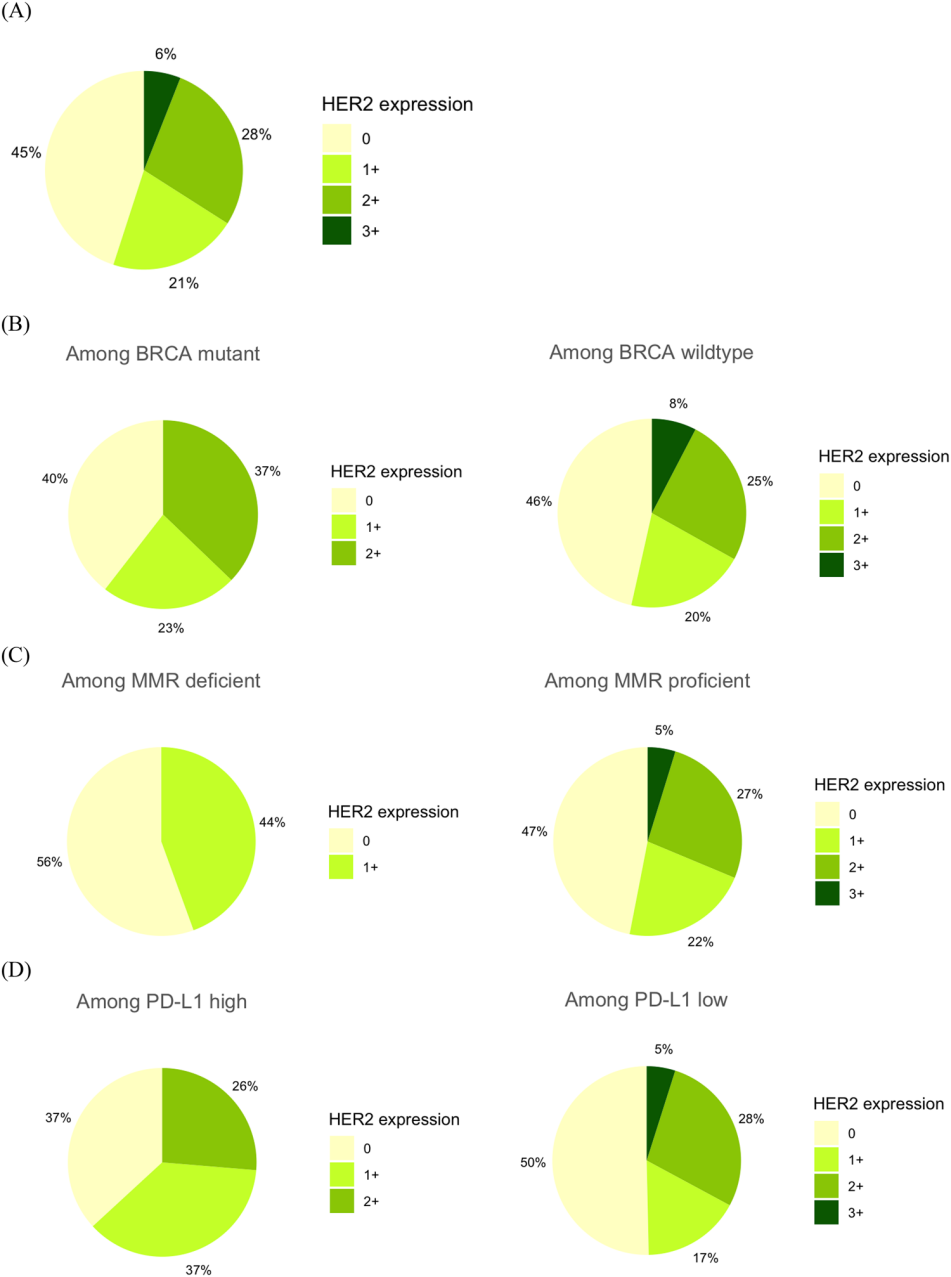
Human epidermal growth factor receptor 2 (HER2) expression in ovarian cancer (OC) stratified by various biomarkers. (**A**) Overall distribution of HER2 expression and that stratified by (**B**) *BRCA*, (**C**) mismatch repair (MMR), and (**D**) programmed cell death ligand 1 (PD-L1) status.

A total of 200 patients were identified, and their demographic information is shown in Table 1. Most of the patients had advanced-stage disease (88%) and high-grade serous histology (76%). *BRCA* mutations were detected in 43 patients (21.5%). Moreover, 42 (21%), 56 (28%), and 12 (6%) patients exhibited HER2 expression scores of 1+, 2+, and 3+, respectively (Fig. 1A). Regarding histological subtypes, the proportions of HER2 3+ scores were 4.6%, 18.8%, 9.1%, and 9.1% in high-grade serous, mucinous, endometrioid, clear cell histology subtypes, respectively (Table 2). No significant differences in HER2 expression regarding histology (p = 0.258) or initial International Federation of Gynecology and Obstetrics staging (p = 0.062) were observed. Furthermore, HER2 expression was stratified based on the status of biomarkers, such as the *BRCA* status, and IHC-based biomarkers, such as MMR and PD-L1, which were tested in 200, 156, and 162 patients, respectively (Fig. 1). HER2 3+ scores were exclusively found in patients with wild-type-*BRCA*, MMR-proficient, and PD-L1-low disease (Fig. 1B–D).

**Table 1:** Patient demographics.

Variable	Patients (n = 200)
Stage	
I	18 (9%)
II	6 (3%)
III	78 (39%)
IV	98 (49%)
Histology	
High-grade serous	152 (76%)
Mucinous	16 (8%)
Clear cell	11 (5.5%)
Endometrioid	11 (5.5%)
Low-grade serous	2 (1%)
Other	8 (4%)
Initial PFI (median, IQR)	14.2 months (9.4–24.9 months)
Distribution based on PFI	
PFI < 6 months	18 (9%)
PFI 6–12 months	59 (29.5%)
PFI > 12 months	123 (61.5%)
BRCA status	
Wild type	157 (78.5%)
Mutant	43 (21.5%)

*PFI* platinum-free survival, *IQR* interquartile range.

**Table 2:** Histology and initial FIGO stage, stratified by HER2 expression levels.

Variables	HER2 0 (n = 90)	HER2 1 + (n = 42)	HER2 2 + (n = 56)	HER2 3 + (n = 12)	P-value
Histology					
High-grade serous	74 (48.7%)	34 (22.4%)	37 (24.3%)	7 (4.6%)	0.2584
Mucinous	5 (31.3%)	4 (25.0%)	4 (25.0%)	3 (18.8%)	
Clear cell	2 (18.2%)	3 (27.3%)	5 (45.5%)	1 (9.1%)	
Endometrioid	5 (45.5%)	1 (9.1%)	4 (36.4%)	1 (9.1%)	
Low-grade serous	1 (50.0%)	0	1 (50.0%)	0	
Other	3 (37.5%)	0	5 (62.5%)	0	
Initial FIGO stage					
I	6 (33.3%)	5 (27.8%)	4 (22.2%)	3 (16.7%)	0.0621
II	2 (33.3%)	0	3 (50.0%)	1 (16.7%)	
III	44 (56.4%)	16 (20.5%)	17 (21.8%)	1 (1.3%)	
IV	38 (38.8%)	21 (21.4%)	32 (32.7%)	7 (7.1%)	

*FIGO* Federation of Gynecology and Obstetrics, *HER2* Human epidermal growth factor receptor 2.

### HER2 expression with respect to tumor NGS findings

**Figure 2:**
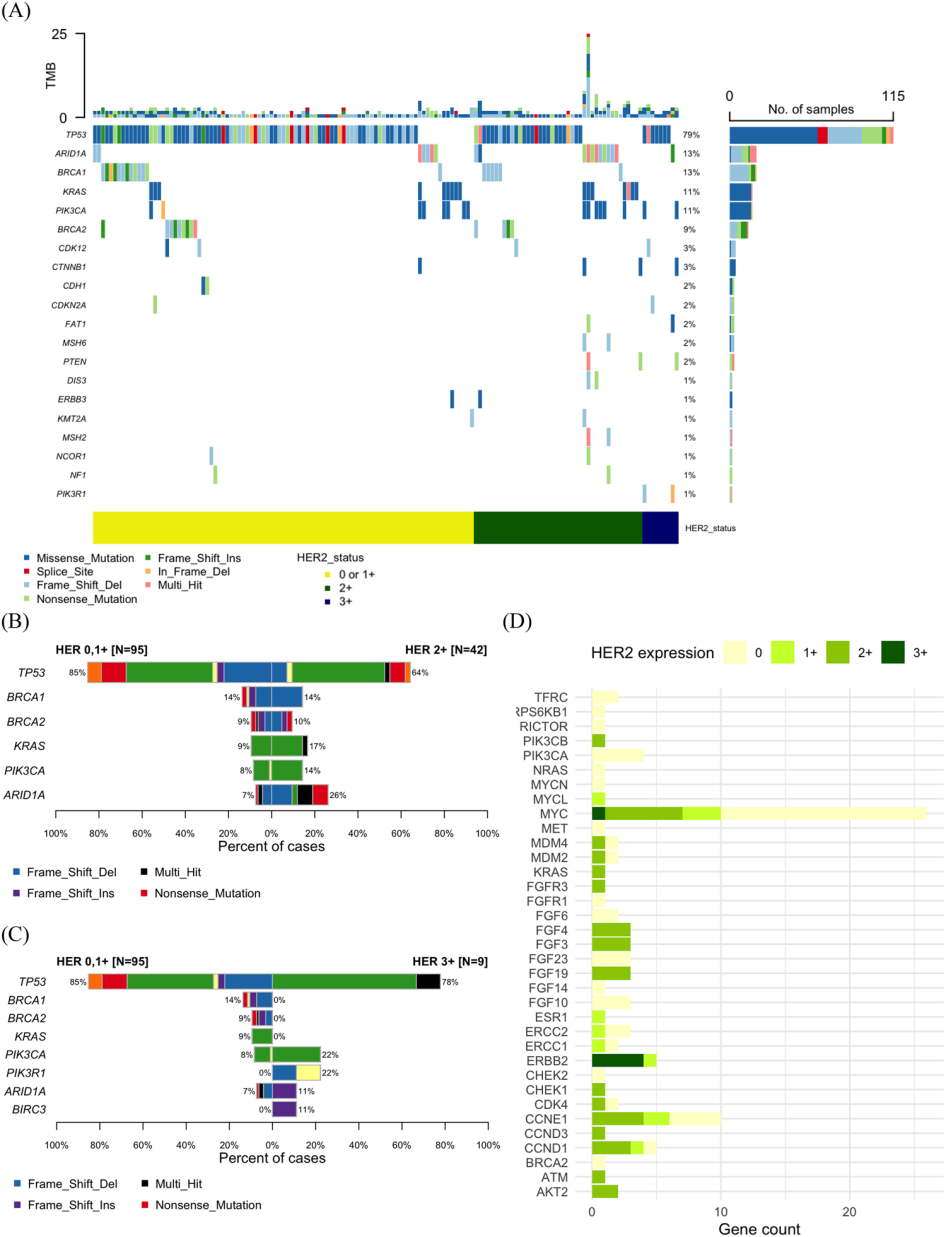
Genomic profile. (**A**) Pathogenic single nucleotide variant alterations in patients with human epidermal growth factor receptor 2 (HER2) scores of 0 or 1+ vs. those in patients with scores of 2+ or 3+. Direct comparison between HER2 0 and 1+ scores vs. (**B**) 2+ and (**C**) 3+ scores. (**D**) Pathogenic variant copy number alterations stratified by HER2 expression.

NGS was performed on tumor samples from 168 of the 200 patients, and the genomic profiles are shown in Fig. 2. Alterations in single nucleotide polymorphism variants with respect to HER2 expression are shown in Fig. 2A. The relative frequency of non-*TP53* mutations was higher in patients with HER2 2+ and 3+ scores. Comparative assessments were performed on the most frequently mutated genes based on HER2 scores of 0 or 1+ versus 2+ (Fig. 2B) and HER2 scores of 0 or 1+ versus 3+ (Fig. 2C). The mutation rate of *TP53* was relatively low, whereas that of *ARID1A*, *KRAS*, and *PIK3CA* was relatively more common in patients with HER2 2+ scores than in those with HER2 0 or 1+ scores (Fig. 2B). Mutations in *PIK3CA* and *ARID1A* were also frequent in patients with HER2 3+ scores (Fig. 2C). Compared with to patients with HER2 0 or 1+ scores, patients with HER2 3+ scores did not exhibit somatic *BRCA1/2* or *KRAS* mutations; however, *PIK3R1* and *BIRC3* mutations were exclusively identified in the latter group (Fig. 2C). Among the subset of five patients who had HER2 3+ expression scores and underwent tumor NGS, four exhibited *ERBB* amplification. The list of actionable mutations in our cohort is shown in Fig. S1.

### Dynamic changes in HER2 expression

**Figure 3:**
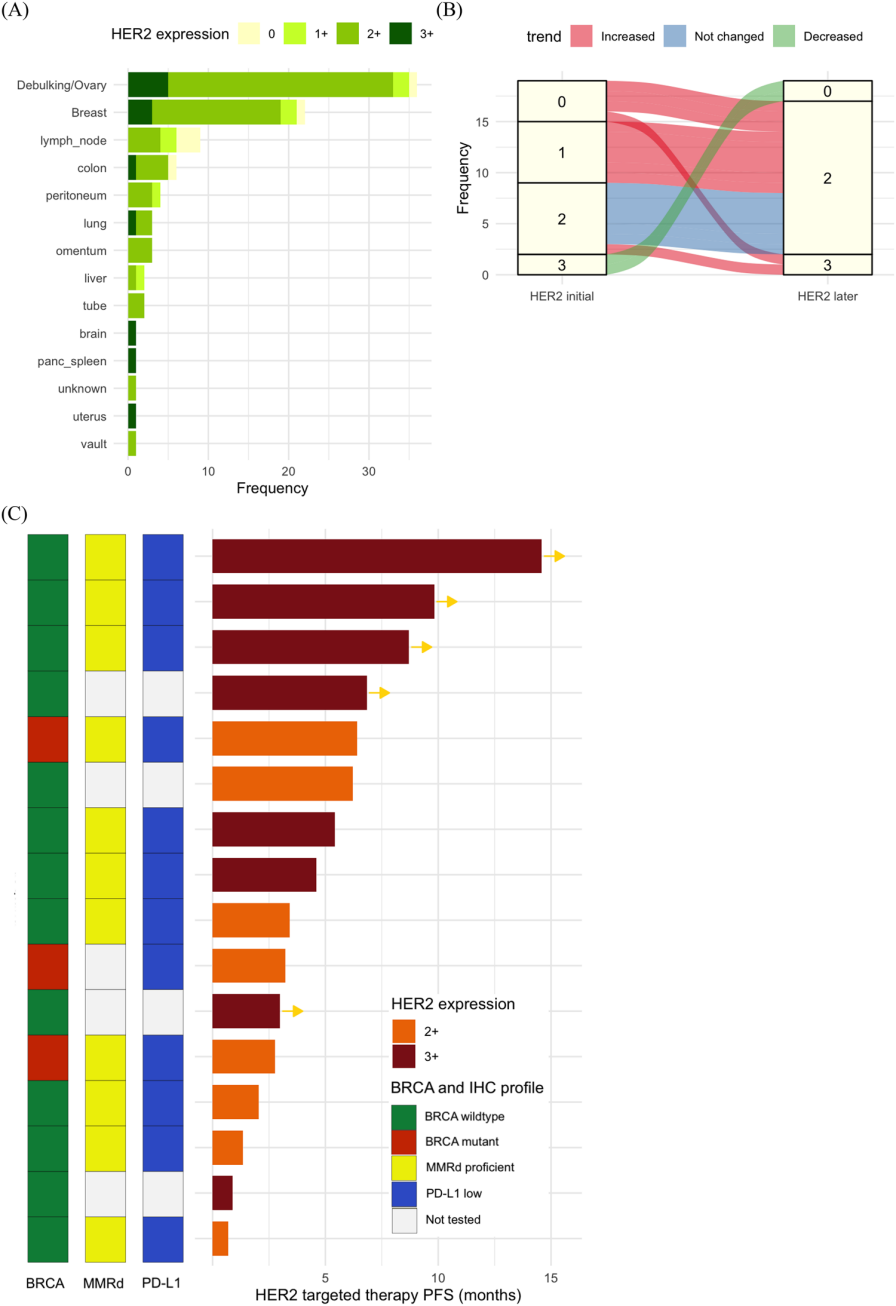
Analysis of multiple ovarian cancer samples and therapy outcomes. (**A**) Human epidermal growth factor receptor 2 (HER2) expression in each anatomic location stratified by HER2 expression. (**B**) Changes in HER2 expression among patients with time-lagged biopsies. (**C**) Swimmer plot showing outcomes of HER2-targeted therapies with *BRCA*, mismatch repair protein (MMR), and programmed cell death ligand 1 (PD-L1) status.

The anatomical distribution of tumors among patients with HER2 2+ and 3+ scores is illustrated in Fig. 3A. Primary debulking specimens from ovaries, breast tissue, and skin biopsies frequently exhibited HER2 2+ or 3+ scores. Among the 19 patients subjected to multiple time-lagged biopsies, HER2 expression was upregulated in 11 patients (57.9%) and remained unchanged in 6 (31.6%) (Fig. 3B). Among those who exhibited upregulated HER2 expression, 10 patients received platinum-based chemotherapy as part of intervening therapy and 4 patients received (poly (ADP-ribose) polymerase) PARP inhibitor. A detailed description of administered therapies and their sequential order is presented in Table S2. Overall, 16 patients with recurrent OC received HER2-targeted therapy. The therapeutic outcomes, HER2 expression, and status of other biomarkers are visually represented in the swimmer plot depicted in Fig. 3C. Durable responses were observed in patients with HER2 3+ scores.

## Discussion

In this study, patients with OC who underwent HER2 testing at Yonsei Cancer Center were investigated. The status of various IHC and genomic biomarkers, results of multiple time-lagged biopsies, and outcomes of HER2-targeted therapy were analyzed. Notably, HER2 3+ scores were exclusively observed in subpopulations characterized by wild-type-*BRCA*, MMR proficiency, and low PD-L1 expression. This subgroup, exhibiting limitations in the efficacy of PARP inhibitors or immune checkpoint inhibitors, signifies an area of unmet therapeutic need, suggesting a potential for HER2-targeted therapy to address such deficiencies in patients with OC. Most patients underwent tumor NGS, which revealed a distinct pattern of single nucleotide variant alterations regarding HER2 expression. Additionally, a notable concordance between HER2 3+ scores and *ERBB* amplification was identified. An analysis of multiple time-lagged biopsies revealed the presence of HER2 overexpression in specific anatomical sites, particularly the ovary and breast. Furthermore, HER2 expression demonstrated frequent upregulation in subsequent time-lagged biopsy samples. This study, constituting the first comprehensive profiling of HER2 in OC to date, provides clinically applicable insights based on real-world data.

Previous studies on HER2 in OC have mainly focused on its positivity rates and association with prognostic factors. The reported HER2 positivity rates in OC have exhibited considerable variability (6.6–39.2%)^16,23^. In this study, we used an IHC-based approach following a protocol used for gastric cancer^17^. Analysis of HER2 expression distribution revealed a 28% rate for HER2 2+ scores and an 8% rate for 3+ scores, both falling within previously reported ranges^16,23^. Our study extends beyond prior studies by revealing the genomic profiles of OC in relation to HER2 status. A notable finding was that HER2 3+ score was exclusively identified in patients with intact *BRCA* status. Nonetheless, a significant limitation of our study is that homologous recombination deficiency (HRD) status was not assessed. Various methods to test for HRD has been reported, which include HRR gene mutation, copy number variation profiles, and signature 3 based on mutational signature analysis^24–26^. Given HRD’s critical role in the management of OC—as it has expended the potential beneficiaries of PARP inhibitor beyond *BRCA* mutation status—future studies on HER2-targeted agents in OC should systemically interrogate HRD with respect to HER2 expression. The outstanding ORR observed in recurrent OC patients in the DESTINY-PanTumor02 (ORR of 63.6% in HER2 3+ score and 36.8% in HER2 2+ score) should be considered within the context of the existing treatment options for OC. Furthermore, similar to previous reports on T-Dxd in HER2-low metastatic breast cancer^8^, the efficacy of HER2-targeted agents in HER2-low OC warrants further investigation, as HER2 1+ or 2+ scores accounted for 49% of our patient population.

Regarding the association between HER2 and prognostic factors, our findings align with those of previous studies, indicating a frequent occurrence of HER2 overexpression in mucinous or clear cell histology subtypes compared to that in high-grade serous histologicalsubtypes^27,28^. In our study, HER2 3+ scores had the highest incidence among mucinous tumors (18.8%), followed by endometrioid (9.1%) and clear cell (9.1%) types, each demonstrating a two-fold elevation compared to high-grade serous tumors (4.6%). Regarding prognostic factors, no specific association was observed between HER2 expression and indicators of poor prognoses, such as an advanced disease stage and initial PFI. The prognostic effects of HER2 expression may vary according to histological subtype, ethnicity, or timing of HER2 testing^29^. In contrast to a previous study on the use of HER2 as a prognostic factor, in which samples from the initial diagnosis or primary surgery were used^30^, our approach involved samples from various time-points throughout the management of OC. Considering the reported change in HER2 expression of 5–10% (up to 20%) following neoadjuvant chemotherapy in breast cancer^15^, the timing of testing might be a confounding factor for OC. Given our frequent testing of HER2 status for clinical trial screening, biopsy samples were also obtained from patients in the setting of recurrence. Despite the inherent challenges in interpreting biopsies from varying time points, we believe that our data represent a real-world depiction of HER2 status testing in patients with OC.

Findings from our analysis of multiple biopsy data highlight the ovarian-specific aspects of changes in HER2 expression during the management of OC. The congruence of HER2 expression across multiple biopsies varied substantially, contingent upon cancer type. Specifically, a consistently robust concordance rate of > 90% has been observed in breast cancer^31^; however, this rate is generally lower (45.5–91.4%) in gastric cancer^32,33^. Although the number of patients who underwent multiple time-lagged biopsies in our study was relatively small, HER2 expression remained unchanged in only 33% of cases. Our findings on HER2 expression in relation to anatomical sites suggest that HER2 overexpression may have a predilection for specific anatomic sites. The ovary from debulking specimens showed a considerable proportion of HER2 2+ or 3+ scores, similar to that in breast biopsy specimens. Patients with initially low HER2 expression frequently show an upregulation in HER2 expression upon metastases to the breast. The notable discordance in the HER2 expression status highlights the potential nuances in HER2 biology and its implication in acquired resistance specific to OC, thereby prompting the imperative need for further translational studies.

Regarding the directionality of the change in HER2 expression, we observed an increase in 66.7% of patients. This predominantly increasing trend differs from that in breast cancer, wherein HER2-negative conversion is equally prevalent, if not more frequent, than positive conversion^34^. Plausible contributory factors encompass both generally applicable elements, such as genetic drift, clonal selection, survival mechanisms, or technical variation^35^, and breast cancer-specific factors, such as the use of trastuzumab, which induces HER2 internalization, decreasing its detectability via IHC^34^. Related to these potential confounders is a significant intrapatient and intermetastasis heterogeneity of HER2 expression, exemplified by the coexistence of HER2-low and HER2-zero metastases in postmortem biopsies^36^. This challenges the current approach of determining HER2-targeted agents based on a single biopsy at a specific time point. Our findings, indicating the frequent upregulation of HER2 expression in time-lagged biopsies, also suggest that repetitive testing could enable the identification of additional patients with OC posed to derive benefit from HER2-targeted therapies. Further studies on the prognostic significance of dynamic changes in HER2 expression are necessary, as a previous study on breast cancer has also suggested that patients with HER2-positive conversion derive substantial benefits from HER2-targeted therapy^34^.

A notable limitation of our study was its retrospective design. The absence of an institutional protocol for HER2 testing may have introduced a selection bias. In addition, the timing and decision to undergo multiple biopsies were at the clinician’s discretion, contributing to a variation in the timing of HER2 testing within our cohort. Furthermore, in a small subset of patients who underwent multiple biopsies, the highest HER2 expression was used for analyses, aligning with the guidelines of the European Society for Medical Oncology for breast cancer, which recommend the use of targeted therapy if HER2 status is positive at least once for patients undergoing multiple biopsies^37^. Lastly, not all patients were subjected to IHC and genomic biomarker analyses, although most patients were. Moreover, our analysis is limited in that it was based on pathologic reports or the analysis of biomarkers was based on pathologic reports or sequencing reports. Further translational studies on HER2 biology and its implications in therapy resistance are imperative. Moreover, prospective studies, particularly those integrated into the framework of clinical trials involving HER2-targeted therapy, offer potential avenues to validate our findings.

In conclusion, we analyzed HER2 expression in OC using comprehensive biomarker analyses, encompassing IHC, germline mutation analysis, and tumor NGS. Our real-world data were based on a sizable cohort, providing valuable insights into the strategic application of HER2 testing OC. We believe that our results will facilitate the identification of optimal candidates for HER2-targeted therapy in OC, particularly within the ongoing landscape of HER2-based trials and future trials involving new-generation HER2-targeted therapies and their associated combinations.

### Supplementary Information

Supplementary Information 1.

Supplementary Information 2.

## Data Availability

The pre-processed mutation raw data that was used for analysis are included as supplementary material.
