# Supplementary material for: Human epidermal growth factor receptor-2 expression and subsequent dynamic changes in patients with ovarian cancer
Source: Sci Rep. 2024 Apr 5;14:7992. doi: 10.1038/s41598-024-57515-y (PMC10997762; doi:10.1038/s41598-024-57515-y)

**Supplementary Material**

Human epidermal growth factor receptor-2 expression and subsequent dynamic changes in patients with ovarian cancer

**Table of contents**

Table S1. Scoring criteria for HER2 expression level.

Table S2. Intervening therapy in patients with increased HER2 expression in time-lagged biopsies.

Figure S1. Actionable mutations based on SNVs in our cohort.

| Score | Expression pattern | Assessment |
| --- | --- | --- |
| 0 | No reactivity or membranous reactivity in <10% of cancer cells | Negative |
| 1+ | Faint or barely perceptible membranous reactivity in ≥10% of cancer cells; cells are reactive only in part of their membrane | Negative |
| 2+ | Weak to moderate complete, basolateral, or lateral membranous reactivity in ≥10% of cancer cells | Equivocal |
| 3+ | Strong complete, basolateral, or lateral membranous reactivity in ≥10% of cancer cells | Positive |

Table S1. Scoring criteria for HER2 expression level.

| Patient | Months between biopsies | HER2 initial | HER2 follow-up | Intervening therapy (line, regimen) |
| --- | --- | --- | --- | --- |
| 1 | 44 | 0 | 2 | 1L Paclitaxel + Carboplatin |
| 2 | 13 | 0 | 2 | 3L Belotecan + Cisplatin, followed by Niraparib |
| 3 | 4 | 1 | 2 | 7L Docetaxel + Cisplatin |
| 4 | 5 | 1 | 2 | 5L Docetaxel + Carboplatin |
| 5 | 6 | 1 | 2 | 5L Gemcitabine + Carboplatin, followed by Niraparib |
| 6 | 9 | 1 | 2 | 4L Paclitaxel + Cisplatin, followed by Niraparib; 5L Gemcitabine |
| 7 | 8 | 0 | 3 | 6L Paclitaxel + Durvalumab |
| 8 | 15 | 2 | 2 | 6L Niraparib; 7L Paclitaxel + Cisplatin |
| 9 | 14 | 1 | 2 | 1L Paclitaxel + Carboplatin |
| 10 | 13 | 1 | 2 | 1L Paclitaxel + Carboplatin |
| 11 | 22 | 2 | 3 | 2L Liposomal doxorubicin + Carboplatin; 3L Paclitaxel + Bevacizumab; 4L Belotecan + Cisplatin; 5L Gemcitabine |

Table S2. Intervening therapy in patients with increased HER2 expression in time-lagged biopsies.

Figure S1. Actionable mutations based on SNVs in our cohort.


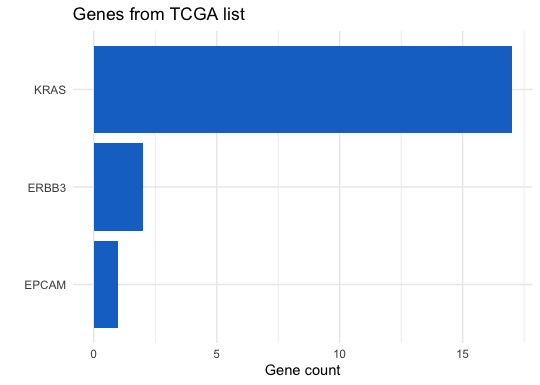

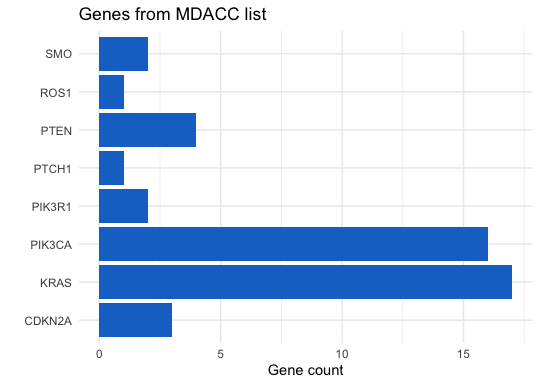

Supplement: Supplementary file 1 — Supplementary Information 1. [file 41598_2024_57515_MOESM1_ESM.docx]
